# Robot-assisted right wedge-shaped sleeve lower bilobectomy with knotless suture anastomosis: a case report

**DOI:** 10.1186/s44215-023-00112-8

**Published:** 2023-11-17

**Authors:** Hironori Oyamatsu, Yusuke Shimura, Ryota Kiriyama, Takehiko Okagawa, Takaki Fujimura, Seijirou Niimi

**Affiliations:** 1 Department of Thoracic Surgery, Okazaki City Hospital, City, Okazaki, Aichi 444-8553 Japan; 2 Department of Respiratory Medicine, Okazaki City Hospital, City, Okazaki, Japan

**Keywords:** Robot-assisted surgery, Squamous cell carcinoma, Wedge resection, Sleeve lobectomy, Knotless suture, Bilobectomy

## Abstract

**Background:**

In bronchoplasty of wedge resections, it is necessary to transect the bronchus at a sharp angle and depth. As a result, anastomoses after wedge resections have the disadvantages of poor visibility and operability. Here, we report a case of right wedge-shaped sleeve bilobectomy that was successfully performed with continuous knotless suturing using robotic assistance.

**Case presentation:**

An 81-year-old male patient was referred for the treatment of a tumor in the right lower lobe, which protruded into the bronchus intermedius. The tumor was diagnosed as squamous cell carcinoma by transbronchial biopsy, cT1cN1M0 stage IIB carcinoma for which surgery was indicated. Because the pulmonary middle lobe artery was involved and a resection margin from the tumor protruding into the bronchial mucosal epithelium was necessary, a right wedge-shaped sleeve bilobectomy was performed. The bronchial anastomosis was performed with robotic assistance. After dissection of pulmonary vessels and interlobes, the upper lobe bronchial bifurcation was transected in a wedged shape, and a lower bilobectomy was performed. The bronchi were sutured continuously with knotless sutures. A continuous suture was performed from the ventral to the caudal side. After suturing to the dorsal side, another continuous suturing was performed from the cranial side. Continuous sutures were made until each thread passed through the other. Pericardial fat was wrapped around the anastomosis.

**Conclusions:**

A better visual field could be obtained owing to robot-assisted surgery, and robotic arms enabled an accurate and safe operation. Furthermore, continuous suturing using a knotless suture made it easier for the sutures to be handled and enabled bronchial anastomosis without assistance.

**Supplementary Information:**

The online version contains supplementary material available at 10.1186/s44215-023-00112-8.

## Background

Bronchoplasty is a useful surgical procedure aimed at complete tumor resection while preserving respiratory function [1, 2]. Bronchoplasty includes tubular or wedge resections. Tubular resections can be used to manage more extensive invasion with better visibility and maneuverability than wedge resection because the central and peripheral bronchi are transected. However, in wedge resections, the bronchus is not transected; although the anastomotic length is short, it is necessary to transect the bronchus at a sharp angle and depth to reduce distortion and kinking after the anastomosis, leading to dehiscence and stenosis. Thus, anastomoses after wedge resections have disadvantages of poor visibility and operability. We report a case of right wedge-shaped sleeve bilobectomy that was successfully performed with continuous knotless suturing using robotic assistance.

## Case presentation

**Fig. 1:**
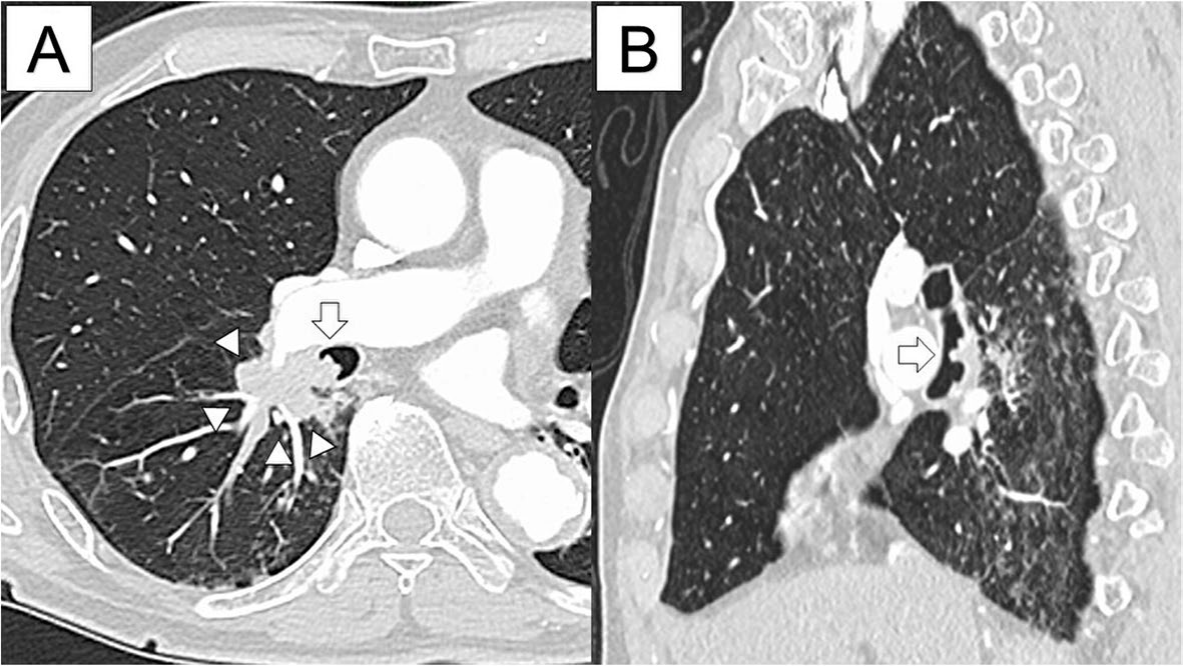
Computed tomography (**A** transverse view, **B** sagittal view) showing a tumor in the right lung S6 (arrowhead), extending continuously from B6 to bronchus intermedius (arrow). The peripheral lung at S6 shows nodular opacities due to obstructive pneumonia

**Fig. 2:**
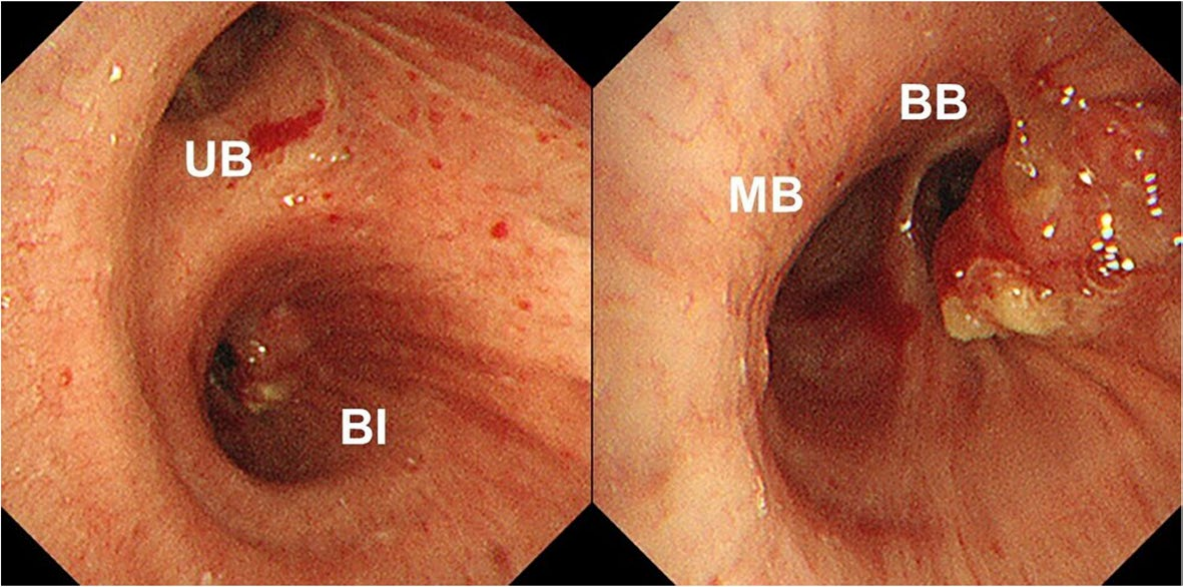
Bronchoscopy showing the tumor extending continuously from B6 to the bronchus intermedius. UB, upper bronchus; BI, bronchus intermedius; MB, middle bronchus; BB, basal bronchus

An 81-year-old male patient was referred for the management of a tumor in the right lower lobe, which protruded into the bronchus intermedius. The tumor was identified on follow-up computed tomography (CT) for a thoracic aortic aneurysm. The CT showed a 2.6-cm tumor in the right lung S6, extending continuously from B6 to the bronchus intermedius. The tumor involved the pulmonary arteries (A4 and A6) and ＃ 12l. The peripheral lung at S6 showed nodular opacities due to obstructive pneumonia (Fig. 1). Bronchoscopic examination revealed a tumor extending from the B6 entrance to the bronchus intermedius (Fig. 2). The tumor was diagnosed as a squamous cell carcinoma by transbronchial biopsy. A positron emission tomography scan revealed an accumulation of SUVmax 13.5 in the right lower lobe tumor involving hilar lymph nodes. There were no indications of mediastinal lymph node or distant metastases. Brain magnetic resonance imaging revealed no metastases. The patient was diagnosed with cT1cN1M0 stage IIB carcinoma, and surgery was indicated.

Because the pulmonary middle lobe artery was involved and the tumor protruded into the bronchial mucosal epithelium of the bronchus intermedius, a right lower bilobectomy was necessary. Bronchoscopy showed that the central edge of the tumor was approximately two rings or less from the right second carina, but the squamous cell carcinoma may have progressed further to the center. We sought to secure the resection margin at a time, and the right second carina was resected partially. We chose wedge-shaped sleeve resection because we considered tubular sleeve resection unnecessary. We planned to perform the bronchial anastomosis with robotic assistance to reduce the invasiveness, secure the visual field, and ensure surgical accuracy. The bronchi were sutured continuously with knotless sutures to shorten the operative time and avoid difficulty in arranging the suture lines.

**Fig. 3:**
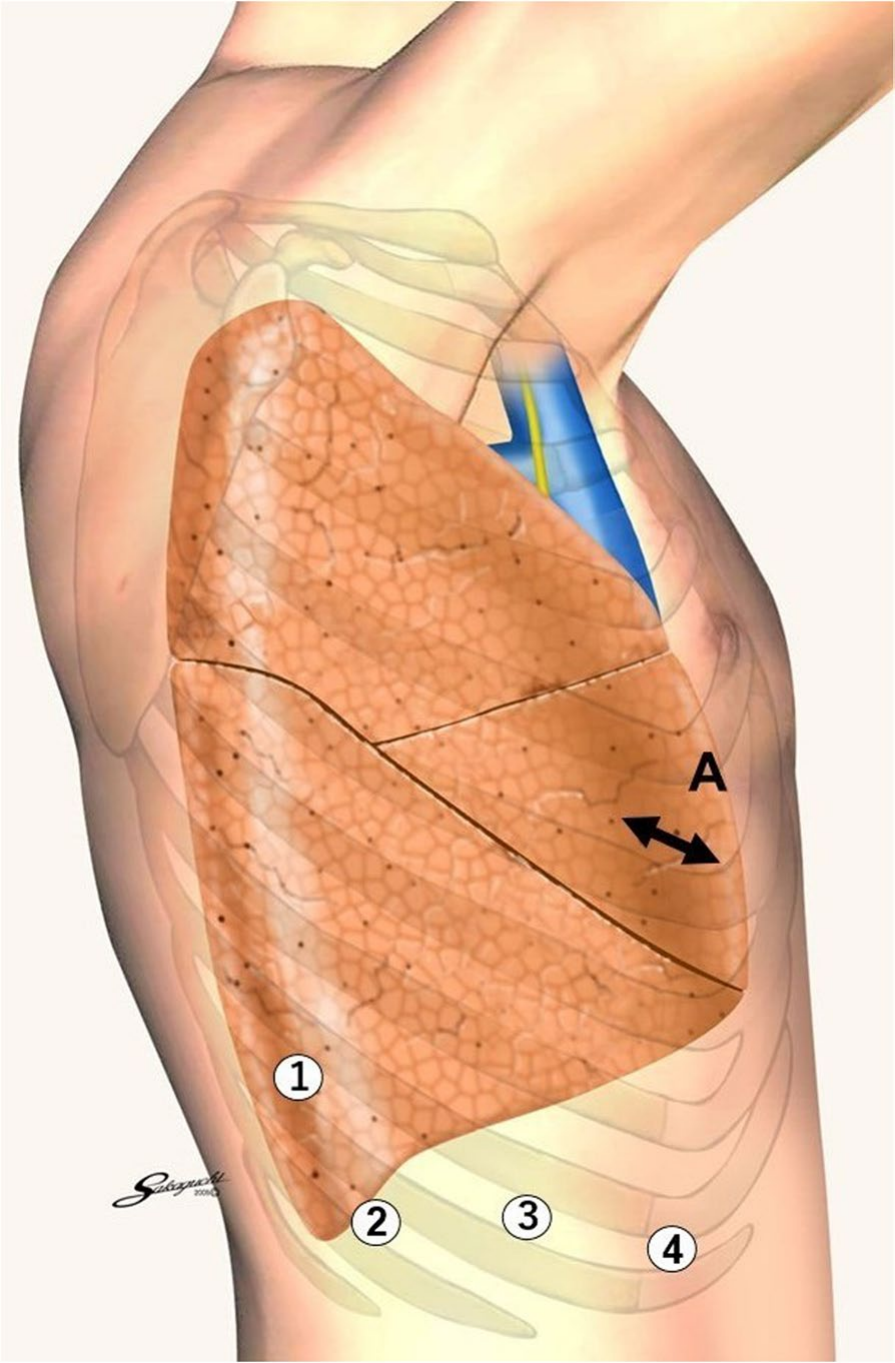
Port placement image. A mini-thoracotomy (for assist port: A) was placed in the anterior axial line in the right fifth intercostal space. A 12-mm port (for fourth robotic arm: 4) was placed on the anterior axillary line in the ninth intercostal space. From there, at equal intervals of 6 cm toward the back, an 8-mm port (for the third robotic arm: 3) was placed in the ninth intercostal space, a 12-mm port (for second robotic arm: 2) was placed in the tenth intercostal space, and an 8-mm port (for first robotic arm: 1) was placed in the ninth intercostal space

**Fig. 4:**
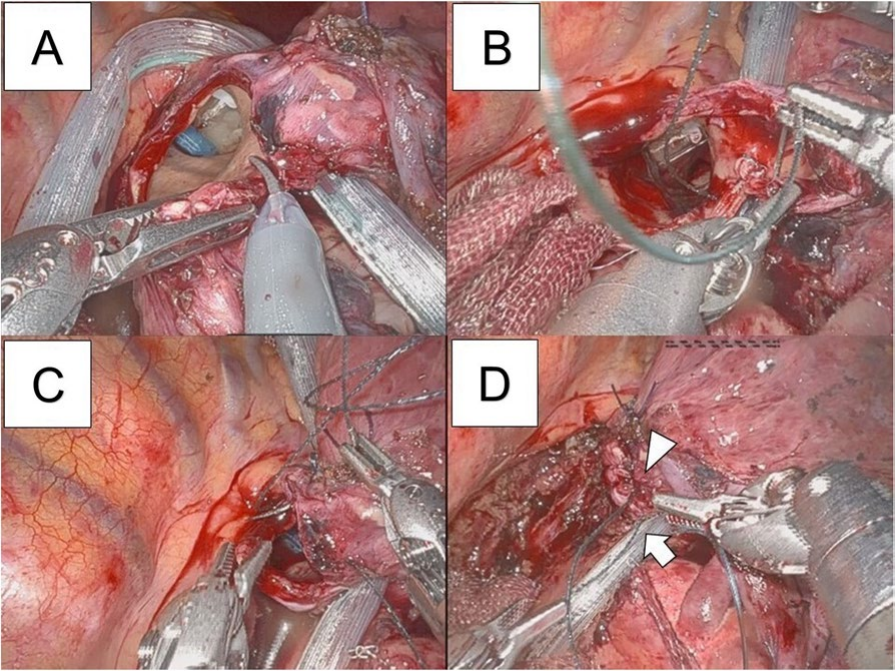
Operative findings. **A** The upper lobe bronchial bifurcation was transected in a wedged shape at a sharp angle and depth. **B** A continuous suture was applied from the ventral side. **C** Another continuous suture was applied from the cranial side. **D** Continuous sutures were made until each thread passed through the other (arrowhead: the suture from the ventral side, arrow: the suture from the cranial side)

A mini-thoracotomy, used as an assist port, was placed in the anterior axial line in the right fifth intercostal space, and a 12-mm port (for the fourth robotic arm) was placed on the anterior axillary line in the ninth intercostal space. From there, at equal intervals of 6 cm toward the back, an 8-mm port (for the third robotic arm) was placed in the ninth intercostal space, a 12-mm port (for the second robotic arm) placed in the tenth intercostal space, and an 8-mm port (for the first robotic arm) placed in the ninth intercostal space (Fig. 3). Each port was connected to the robotic arm of the da Vinci® Xi™ Surgical System (Intuitive). A tip-up fenestrated grasper was used for the first robotic arm. Cadiere forceps were used for the second robotic arm, and a large needle driver was used during bronchial anastomosis. A camera was used for the third robotic arm. Maryland bipolar forceps were used for the fourth robotic arm, Monopolar curved scissors were utilized during bronchial dissection, and a large needle driver was utilized during bronchial anastomosis. Intrathoracic surgery was performed using CO2 gas insufflation at 8-mmHg pressure. The interlobar fissures between the upper and lower lobes and the upper and middle lobes were tunneled, dissected, and separated. The inferior pulmonary vein, middle lobe pulmonary vein, A5, and peripheral pulmonary arteries from the A2b bifurcation were dissected. The upper lobe bronchial bifurcation was transected in a wedged shape, and a lower bilobectomy was performed. An intraoperative frozen section of the resected bronchial stump was negative, and anastomosis of the wedge-shaped dissection was initiated. A continuous suture was performed from the ventral to the caudal side using 3-0 V-Loc™ 180 (Medtronic). After suturing to the dorsal side, continuous suturing was performed using another 3-0 V-Loc™ 180 from the cranial side. Continuous sutures were made until each thread passed through the other. Pericardial fat was wrapped around the anastomosis. After piercing the pericardial fat with two threads, the two threads were ligated to suture the pericardial fat onto the anastomosis (Fig. 4, Additional files 1–4).

**Fig. 5:**
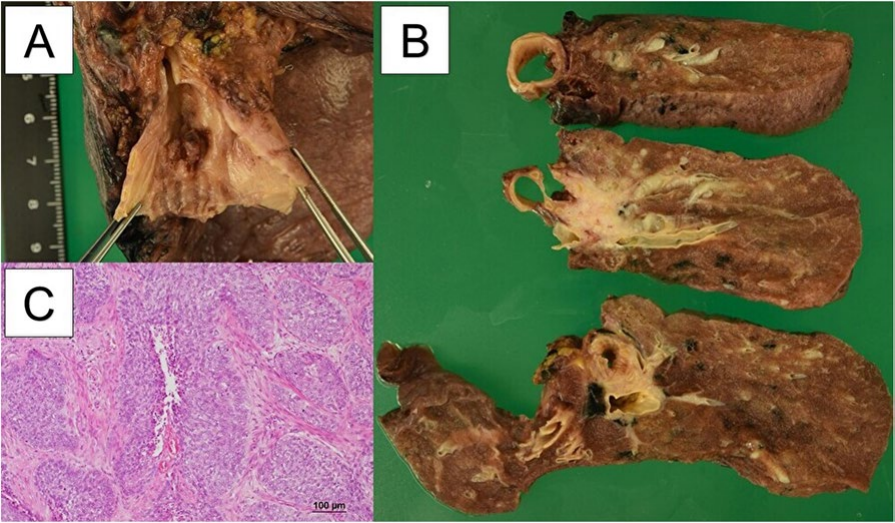
**A** Although the leading edge of the tumor extends to the vicinity of the bronchial stump, the stump is negative for cancer. **B** Gross findings showing tumors involving the bronchi and vessels and pus in the peripheral lung. **C** Proliferation of high-grade dysplastic squamous epithelial cells in the alveolar foci with central necrosis

Histopathologically, although the leading edge of the tumor extended to the vicinity of the bronchial stump, the stump was negative. Proliferation of high-grade dysplastic squamous epithelial cells was identified in alveolar foci with central necrosis (Fig. 5). No keratinization was observed. Staining for p40 was positive, whereas TTF-1 and synaptophysin were negative. Therefore, it was considered that the lesion was a non-keratinizing squamous cell carcinoma. ＃ 12l showed metastatic changes.

**Fig. 6:**
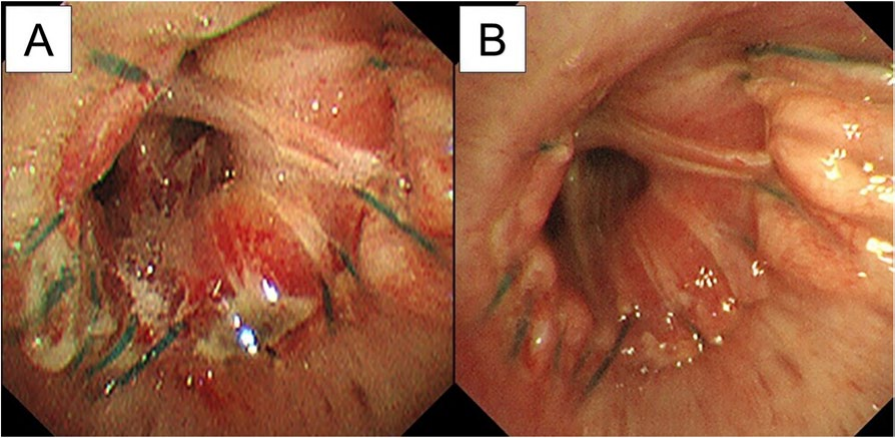
Bronchoscopy at 1 week (**A**) and 1 month (**B**) after the operation showing anastomotic site healing

The postoperative course was uneventful, and a bronchoscopy performed 1 week and 1 month after the operation confirmed that healing of the anastomotic site had occurred (Fig. 6).

## Discussion and conclusion

Robot-assisted thoracic surgery has been recently introduced. The disadvantages of robot-assisted surgery include longer operative times, higher medical costs owing to the use of special instruments, and a lack of tactile feedback, which must be compensated for by visual feedback [3, 4]. However, it also has several advantages. First, the operator sits down during the surgery, which reduces mistakes caused by fatigue during long and complicated surgeries, improving surgical safety. Second, the robotic arm imitates the movement of the operator’s hand, and hand tremors are corrected. The connected instruments are small, and the joints move well; thus, surgery can be performed accurately and freely in small spaces. Third, tissues are easily identified on an enlarged three-dimensional field of view [3, 4]. In addition, by infusing CO2 into the thoracic cavity, the mediastinum and diaphragm are pushed aside, and a better visual field is ensured [4]. Robot-assisted surgery is considered suitable for bronchoplasty as it is less invasive, provides a better visual field, and enables more accurate needle placement. The prognosis of robot-assisted sleeve lobectomy is also reported to be comparable to that of thoracotomy or thoracoscopic surgery [1].

The suturing methods for bronchoplasty include the use of interrupted or running sutures. Conventionally, bronchial anastomoses with interrupted sutures were often performed; however, in recent years, bronchial anastomoses with continuous sutures have been introduced. Interrupted suture anastomoses require many suture threads, and the technique is time-consuming and difficult to perform [2]. The disadvantage of continuous sutures is that if they are cut, the entire suture will be incomplete. However, it has the advantage of being able to secure an anastomosis in a limited field of view and a narrow space because it omits the difficulty in the arrangement of suture lines, shortens the operative time because there are fewer ligatures, and distributes tension over the entire area [2, 5]. The knotless suture used in this patient had one-way barbs on the entire circumference; therefore, it could continue to hold the tissue without loosening, making it easier to handle the thread. The operative time can also be shortened by fixing the suture through the loop without ligating the beginning of the suture. Although in robotic surgery the robotic arm may be difficult for the assistant to maneuver, making it difficult to arrange suture lines, continuous suturing with a knotless suture allows the operator alone to perform bronchial anastomoses.

We performed a robot-assisted right wedge-shaped sleeve bilobectomy using continuous knotless suturing. Although transection of the bronchus and suturing techniques was performed in a narrow space, a better visual field could be obtained owing to robot-assisted surgery and CO2 infusion of the thoracic cavity, and robotic arms enabled an accurate and safe operation. Furthermore, continuous suturing using a knotless suture made it easier to handle the sutures because there was no need to maintain traction on the thread to bring the bronchi closer together, enabling bronchial anastomosis without assistance. However, because fixation by loops may be inferior to fixation by ligatures in terms of tissue retention at the beginning of suturing, frequent suturing may be required at that time. In addition, as there was no tactile feedback, it was necessary to carefully apply tension using sufficient visual feedback when pulling on the thread to approximate the bronchi. Looking back at the surgery, there were times when the bronchi were grasped for more accurate needle placement during the anastomosis. No problems were experienced with the grasping. Since robot-assisted surgery enables accurate and flexible manipulation, it is better to be careful not to damage the bronchial wall.

## Supplementary Information

**Additional file 1.** The movie of Figure 4A.

**Additional file 2.** A-C The movie of Figure 4B.

**Additional file 3.** A-C The movie of Figure 4C-D.

**Additional file 4.** The movie of pericardial fat wrapping around the anastomosis and sutured.

## Data Availability

Not applicable

## Abbreviation

CT: Computed tomography
